# ‘Condoms are hard to get by’: access to HIV prevention methods during lockdown of the COVID-19 epidemic in eastern Zimbabwe

**DOI:** 10.1080/16549716.2023.2206207

**Published:** 2023-05-03

**Authors:** Morten Skovdal, Tanyaradzwa Maunzagona, Freedom Dzamatira, Phyllis Magoge-Mandizvidza, Rufurwokuda Maswera, Brian Kumbirai Moyo, Constance Nyamukapa, Simon Gregson

**Affiliations:** a Department of Public Health, University of Copenhagen, Copenhagen, Denmark; b Manicaland Centre for Public Health Research, Biomedical Research and Training Institute, Harare, Zimbabwe; c HIV and TB Unit, Ministry of Health and Child Care, Harare, Zimbabwe; d Department of Infectious Disease Epidemiology, Imperial College London, London, UK

**Keywords:** COVID-19, pandemic response, HIV prevention, condoms, Zimbabwe

## Abstract

**Background:**

In the early phase of the coronavirus disease 2019 (COVID-19) pandemic, health services were disrupted worldwide, including HIV prevention services. While some studies have begun to document the effects of COVID-19 on HIV prevention, little has been done to qualitatively examine how lockdown measures were experienced and perceived to affect access to HIV prevention methods in sub-Saharan Africa.

**Objectives:**

To explore how the COVID-19 pandemic was perceived to affect access to HIV prevention methods in eastern Zimbabwe.

**Method:**

This article draws on qualitative data from the first three data collection points (involving telephone interviews, group discussions, and photography) of a telephone and WhatsApp-enabled digital ethnography. Data were collected from 11 adolescent girls and young women and five men over a 5-month period (March–July 2021). The data were analysed thematically.

**Results:**

Participants reported widespread interruption to their condom supply when beerhalls were shut down as part of a nationwide lockdown. Restrictions in movement meant that participants who could afford to buy condoms from larger supermarkets or pharmacies were unable to. Additionally, the police reportedly refused to issue letters granting permission to travel for the purpose of accessing HIV prevention services. The COVID-19 pandemic was also described to obstruct the demand (fear of COVID-19, movement restrictions) and supply (de-prioritised, stock-outs) for HIV prevention services. Nonetheless, under certain formal and informal circumstances, such as accessing other and more prioritised health services, or ‘knowing the right people’, some participants were able to access HIV prevention methods.

**Conclusion:**

People at risk of HIV experienced the COVID-19 epidemic in Zimbabwe as disruptive to access to HIV prevention methods. While the disruptions were temporary, they were long enough to catalyse local responses, and to highlight the need for future pandemic response capacities to circumvent a reversal of hard-won gains in HIV prevention.

## Introduction

Efforts to curb new SARS-CoV-2 infections and coronavirus disease 2019 (COVID-19) related deaths have resulted in a global and unprecedented public health response. The COVID-19 pandemic and its associated preventative measures have disrupted health services worldwide, including those designed to prevent the spread of HIV [1–3]. Although Zimbabwe started to close its borders and schools on 23 March 2020, the first COVID-19-related national lockdown came into effect a week later and lasted until 3 May. Periodic lockdowns ensued in response to spikes in new daily cases (January to February and July to September 2021) and included restrictions on intercity movements and public gatherings, curfews, limited opening hours of essential shops, and closure of informal markets and beerhalls. How did these lockdown periods affect access to primary HIV prevention methods? We explore this question from the perspectives of people living in high HIV prevalence and low resource settings of east Zimbabwe.

Prior research on the impact of COVID-19 on HIV prevention is limited, and what is available paints a mixed picture. Studies from the United States (US) report widespread closure of, and disruption to HIV prevention services, challenging access, particularly for disenfranchised groups [4,5]. A qualitative study by Zapata et al. [6] documents how young sexual minority men experienced limited and disrupted access to HIV testing, HIV pre-exposure prophylaxis (PrEP), and HIV post-exposure prophylaxis (PEP). However, where HIV prevention services were adapted to the COVID-19 pandemic, for instance, by developing a telemedicine infrastructure [7], a different story emerges. A health record analysis of PrEP, HIV, and STI visits at eight sexual health clinics in the US found telemedicine adaptations to increase PrEP uptake among men [8]. In Brazil, Dourado et al. [9] noted a continuation of HIV prevention service use among adolescents owing to telemedicine adaptations.

Research from Southern Africa is also mixed. In South Africa, a study found evidence of reduced access to condoms during the COVID-19 pandemic [10], while early programmatic observations noted an increase in the number of pregnant women on PrEP missing their follow-up appointments [11]. Women were found to fear COVID-19 and contact with health facilities [11]. A qualitative study noted numerous obstacles to the prevention of mother-to-child transmission among migrants during the South African COVID-19 pandemic, including overworked health facilities, border closures, and a lack of documentation, as well as xenophobia against migrant HIV populations [12]. For female sex workers, however, a different picture emerges. Despite the COVID-19 pandemic, the number of female sex workers in eThekwini, South Africa, either initiating or staying on PrEP increased in the period 2016–2020 [13]. Similar observations have been made in urban programmes in Zimbabwe, where the uptake of PrEP among female sex workers increased dramatically as a result of adaptations that were made to their delivery, shifting services from clinical settings towards community/home-based, peer-led PrEP services [14].

In this study, we build on this small body of work and explore how people in eastern Zimbabwe experienced lockdown measures in relation to access to HIV prevention methods as well as the circumstances that helped some adapt to the disruption in access. In doing so, we respond to calls for detailed and contextualised analyses to assess how coverage of different HIV prevention methods and services were affected by COVID-19 and why [15].

## Methods

This qualitative and exploratory study forms part of a larger open-cohort research project, which seeks to capture the impact of COVID-19 at different stages of the epidemic on sexual risk behaviour and HIV infection in Zimbabwe. Underpinned by digital ethnography, this qualitative study followed a small cohort of individuals over a 12-month period using WhatsApp-enabled smartphones. The research project was reviewed and approved by the Imperial College Research Ethics Committee (Ref. 20IC6436), and the Medical Research Council of Zimbabwe (Ref. MRCZ/A/2703). Approval was granted on the condition that we protected participants’ identities. Therefore, their names have been replaced with pseudonyms. All participants provided informed consent to participate at the onset of the study and for each data collection activity.

### Study location and participants

The study was conducted in the Manicaland Province of eastern Zimbabwe. Manicland province is characterised by high levels of poverty and HIV. The HIV prevalence in the adult population of the province was in 2020 estimated to be 10.2% (95% CI 8.6–11.9) [16]. Because the study was conducted via telephone, there were no logistical reasons to limit the geography of the study to specific sites within the province. Drawing on an open-cohort dataset, we recruited participants who were at a heightened risk of acquiring HIV from a mix of settings, including rural subsistence farming areas, small towns, large-scale agricultural estates, and high-density city suburbs. These settings have given rise to different preconditions for experiencing the social dynamics of HIV and COVID-19. The inclusion criteria included self-reported sexual debut as well as sexual activity within the last six months. We also restricted the age groups to reflect HIV risk. Women had to be between the ages 18–24, whilst men could be in the 20–54 age group. We also decided that half of the women should have self-reported engagement in transactional sex. Participants meeting the criteria were randomly sorted and added to a list for the study team to contact.

We recruited eighteen participants, representing young women (*n* = 6), young women engaged in transactional sex (*n* = 6), and men (*n* = 6). All participants had some level of secondary education. Two participants lived with HIV, and two-thirds of the participants were married. The average age was 27. The oldest participant was age 46, whilst the youngest was aged 21. Six of the female participants were unemployed and took care of their family. Other participants worked either in the informal sector or as subsistence farmers.

Participants were introduced to the study and its longitudinal nature via telephone. They were informed that we would lend them a bespoke camera-phone with WhatsApp installed for a 12-month period, and that we would provide them with airtime. They were invited into two different WhatsApp groups; one with the researcher, and one for their sub-group (i.e. men, AGYW, women engaged in transactional sex), through which a mix of synchronous and asynchronous interviews, group discussions, photography and audio recording exercises were facilitated. Two participants dropped out of the study after the first activity (telephone interviews), for different reasons. One young woman struggled to use the mobile telephone and experienced significant network challenges. Another young woman who engaged in transactional sex become overwhelmed with the care of a family member.

### Data collection and analysis

**Figure 1.**
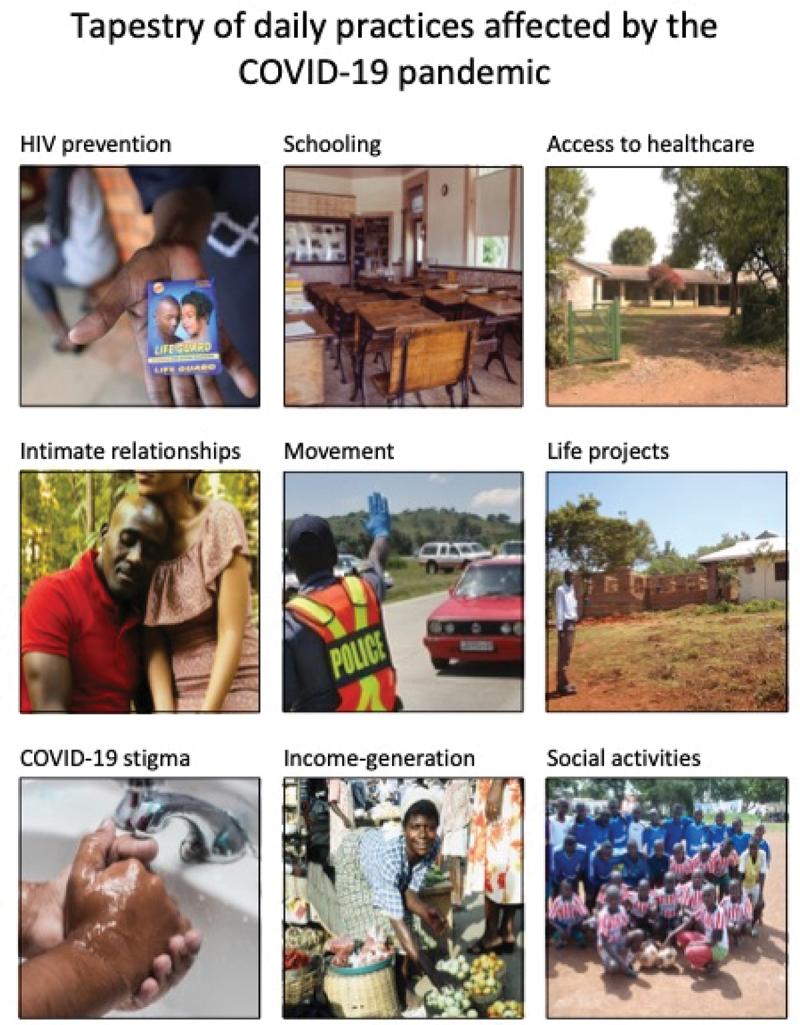
Visual used for ‘Tapestry of daily life’ exercise.

This study draws on data generated between March and August 2021 through the first three activities of the longitudinal study. Data were collected by TM, a Shona-speaking qualitative social scientist. She was unknown to the participants prior to the study. The data collection activities included telephone interviews (March–April), ‘Tapestry of daily life’ group discussions (May-June) and ‘Picture this’, a Photovoice [17] exercise (July–August). The telephone interviews were semi-structured and steered using an interview guide covering the following topics: life during COVID-19, responses to COVID-19, dating and sexual encounters, and engagement with HIV prevention methods. An initial thematic network analysis [18] of the interviews revealed nine life practices that were affected by COVID-19. These nine impacts were visually represented in a tapestry using images identified by the researchers (see Figure 1), steering the ‘Tapestry of daily life’ exercise. This exercise involved a synchronous WhatsApp group discussion on the nine impacts on daily life. The aim of this exercise was to explore the impacts at group and community levels, akin to a focus group discussion steered by a visualisation. The group discussions were exported into a word document and summarised by TM.

Photovoice, a methodology that allows participants to visually identify and represent issues of importance to them [17,19], was introduced to participants through the three WhatsApp sub-groups, in which ethical considerations of the exercise were also discussed. These included how to obtain consent from subjects and alternative ways of capturing a story to minimise ethical dilemmas. Over the course of two weeks, participants took photographs representing life during COVID-19; the good things, the difficulties and the things that need to change. In a one-to-one and asynchronous WhatsApp chat with the researcher, participants were encouraged to select two pictures that show someone or something that is important for them, two pictures that show something or someone who puts them at risk of HIV, and two pictures that show something or someone who protects them from acquiring HIV. The researcher invited participants to write a story – in the WhatsApp chat – for each of their six photos, explaining why they wanted to share that photo, the story it tells, how this story relates to their lives, and the lives of their peers. The participants shared more or fewer photos than encouraged, amounting to a total of 61 images. All interview transcripts, WhatsApp transcripts of group discussions, pictures, and their accompanying stories as well as researcher notes, were imported into NVivo 12 for thematic coding and analysis. The different types of data were treated as a single data corpus and subjected to thematic network analysis, following the steps outlined by Attride-Stirling [18]. This involved MS coding all the data. Codes were organised in networked hierarchies, through what NVivo 12 refers to as ‘child nodes’, ‘parent nodes’ and ‘grandparent nodes’. This process generated 15 ‘grandparent’ nodes, covering a broad range of topics, such as experiences of lockdown measures, participation in the COVID-19 response, COVID-19 stigma, and the financial impacts of lockdown. In this article, we report on one of the 15 ‘grandparent’ nodes, namely the global theme of (breaking) barriers in access to HIV prevention methods. In preparation for this manuscript, the 17 codes constituting this theme were clustered into 11 basic themes, which in turn were grouped together under three more interpretative organising themes that constitute headline findings (see Table 1). Our themes and the interpretation of higher-order themes were informed by the discipline of community health psychology, heightening our attention to community responses to COVID-19. We present and further analyse the 11 basic themes using the structure of the thematic network.

**Table 1.** Thematic network of basic and organising themes.

Basic themes	Organising themes	Global Theme
Closure of beerhalls where condoms are available	1. ‘Locked-out’ and ‘locked-in’ from lockdown	When pandemics collide: Local impacts and responses to HIV prevention in the face of national responses to COVID-19
Closure of shops and restricted opening hours of pharmacies selling condoms		
Movement restrictions prevent access		
COVID-19 induced poverty heighten barriers to access		
Clinics not getting re-stocked with HIV prevention methods	2. Disruptions and fear prohibiting access to HIV prevention services	
HIV prevention services not prioritised by healthcare staff		
Stigma and fear of COVID-19 prevent people from accessing HIV prevention services		
Some community health workers play an active role in distributing condoms	3. ‘Behind the scenes’ tactics to access HIV prevention methods	
Knowing the right people can give informal access to condoms		
Women visiting health services for maternal and child health reasons use it as an opportunity to access condoms		
Sex workers draw on their relationships with local healthcare providers to access HIV prevention methods		

## Results

All participants spoke in detail about how strict lockdown measures affected access to HIV prevention methods. They either spoke from personal experiences or verbalised local perceptions.

### ‘Locked-out’ and ‘locked-in’ from lockdown

In Zimbabwe, condoms are freely available from beerhalls. According to our participants, it is also the place where most men access condoms. When beerhalls closed in March 2020 (they re-opened for vaccinated patrons at the end of November 2020), a significant barrier to condom access was introduced. Almost all participants spoke about the breakdown in condom access as result of beerhall closures. People were ‘locked-out’ of the venues where condoms were ordinarily obtained. One participant said: ‘Many people get condoms in beerhalls but since these were closed, there was nowhere to get them’. Similar accounts came from people who used to access condoms from shops, either because they too were periodically shut down or because travel restrictions made it difficult for them to visit the shops.

‘The challenge we are facing is that I usually use condoms and it is difficult for me to travel to the places where I buy them. Before COVID, shops and tuck shops were open and I would easily run and buy there, now it is difficult because they are closed. So when my partner comes we end up having unprotected sex because I cannot let her go back without having sex as she would have come for sex. Even the bars from which we could get they are also closed.’ John, age 38, live in a high-density city suburb

John also alludes to a potential consequence of this breakdown in condom access, namely unprotected sex. Whilst a few participants shared similar experiences, others reported abstinence as the alternative method of HIV prevention.

Hospitals and clinics were also often talked about as a place to access HIV prevention methods; although, for some, these were unusual and challenging places. John went on to explain that ‘it is embarrassing for us to [go to the hospital and] say that we want condoms.’ During the initial lockdowns, movement was limited to 5 km from people’s homes. Being ‘locked-in’ by restrictions to movement proved a challenge for people with no health facilities in a 5 km radius. Jane, a 25-year-old woman, said, ‘During lockdown people were unable to get their PrEP or condoms because movement was restricted.’ Another participant, Alice, recounted through photography the mobility challenges she faced in accessing PrEP during lockdown, including the need to obtain a mandatory clearance letter from the police, permitting her to travel to a hospital to access PrEP.

**Figure 2.**
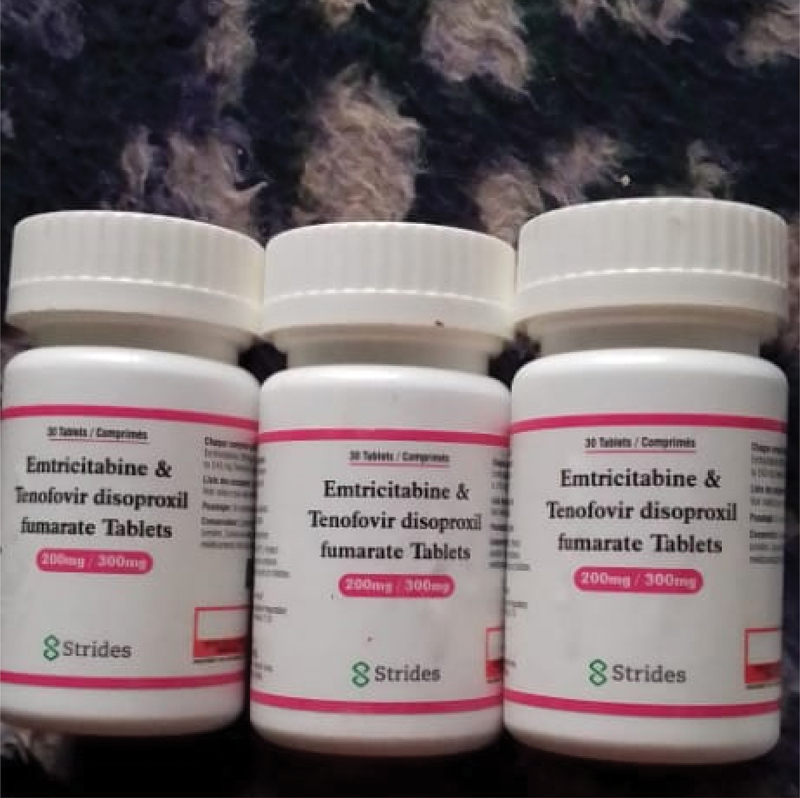
‘PrEP’.

‘The picture (Figure 2) shows PrEP tablets and they prevent me from acquiring HIV. The laws implemented by the government made it difficult for us to get methods of preventing HIV because people were not allowed to move around. Even if one wanted to go to the hospital, a travel permit letter was required which showed the importance of where you were travelling to.’ Alice, age 24, live in a high-density city suburb

Some participants perceived such clearance letters to be impossible to obtain for the purpose of accessing HIV prevention services, as exemplified by Vincent: ‘Letters to move around were not being given for reasons, such as getting condoms and PrEP or VMMC.’ Vincent also explained that many people feared telling the police they needed a letter granting them access to travel for the purpose of accessing HIV prevention methods. Anaisha described how only health emergencies would be allowed to travel to the hospital, limiting young girls’ access to condoms.

’Young girls engaged in unprotected sex because the hospitals where they used to collect condoms from were inaccessible. Only people with emergency cases were allowed to travel to hospitals.’ Anaishe, age 25, live in a high-density city suburb

Many participants spoke about the impact of COVID-19 measures on people’s livelihoods. On a few occasions, the economic impact of COVID-19 was linked to access to HIV prevention services. For instance, in a group discussion with the men, COVID-19 induced poverty was said to indirectly affect HIV testing and prevention services by limiting people’s means of transport.

‘Those that needed money for travelling to go and get tested for HIV or get other prevention methods such as condoms and PrEP could not afford to do so because all the money had to be for food.’ Samuel, age 27, live on a large-scale agricultural estate

Broadly, participants in the study reported lockdown-related barriers to their access to HIV prevention methods from a range of settings.

### Disruptions and fear prohibiting access to HIV prevention services

The participants, however, also spoke about how the COVID-19 pandemic interfered with the provision of more formal HIV prevention services. A couple of participants articulated a perception that the COVID-19 pandemic had affected the supply and delivery of HIV prevention methods to clinics and hospitals.

‘It has become difficult to access things like PrEP and condoms in the hospitals due to the limited resources […] sometimes the hospitals run out of things like condoms and PrEP.’ Maria, age 24, live in a high-density city suburb

Of particular concern for our participants was the perception that health facilities had run out of HIV prevention methods, and therefore were not open for provision of HIV prevention services, as explained by Anaishe:

‘At the hospital we would wait outside as we were not allowed to get in. When you got there they would ask “How may we help you?” and one would respond that I have come to collect condoms or PrEP tablets. They would respond that we do not have condoms, medicine or even PrEP and we are also not assisting anyone as we are in lockdown. We would return home with nothing.’ Anaishe, age 25, live in a high-density city suburb

For others, hospitals were perceived to simply not prioritise provision of HIV prevention services. Rapid screenings were done at the gates of health facilities to limit the number of people entering, and only people seeking treatment in relation to COVID-19 illness, maternal and child health, as well as medical emergencies were prioritised. This is described by Albert who also pointed to a solution that grew organically out of this problem; the distribution of condoms by community health workers.

‘It was not easy to access condoms during the pandemic as the clinics limited the number of people coming in. You had to be screened outside the gate. If your request was to get condoms, you would not be prioritized. Medical emergencies had to be attended to first. However, they came up with the solution that community health workers stayed with the condoms at their houses and you could access them from their houses, to try and ease pressure at clinics.’ Albert, age 28, live on a large-scale agricultural estate

Some participants described a general fear of COVID-19 in the early months of the pandemic. A fear that was prevalent both among healthcare providers (before they were vaccinated) and health service users. This fear, and the knowledge that people affected by COVID-19 would congregate at clinics and hospitals, resulted in some people avoiding hospitals, restricting their access to HIV prevention methods. According to our participants, the COVID-19 pandemic, in different ways, weakened the formal provision of HIV prevention services.

### ‘Behind the scenes’ tactics to access HIV prevention methods

Although lockdown measures and the COVID-19 pandemic were predominantly described as obstructing access to HIV prevention methods, some people were, under certain circumstances, able to either navigate the system or negotiate access through local social structures. Above, Albert described the role of community health workers in distributing condoms. Other participants spoke about the role of community health workers in bridging HIV prevention services (supply) with demands for HIV prevention methods, as exemplified by Joyce.

‘The village health worker helped me access HIV prevention methods like condoms and PrEP. During this COVID-19 era, I see him without any struggle and I still receive assistance. The other community members are getting the same assistance from the village health worker. Some fear to go to the village health work because they fear they might contact COVID-19 virus.’ Joyce, age 22, live in a small town

Joyce, however, also alluded to how community health workers, much like hospitals and clinics, were by some considered proxies for COVID-19 infection, limiting their appeal. Others described that they had no relationship with the local community health worker, and therefore did not feel comfortable approaching them for condoms. Knowing the right people and personal connections seem to matter. Aneni, through a photo of a closed beerhall, described how knowing the owner or staff at a beerhall could grant you access to condoms (getting ‘locked-in’).

**Figure 3.**
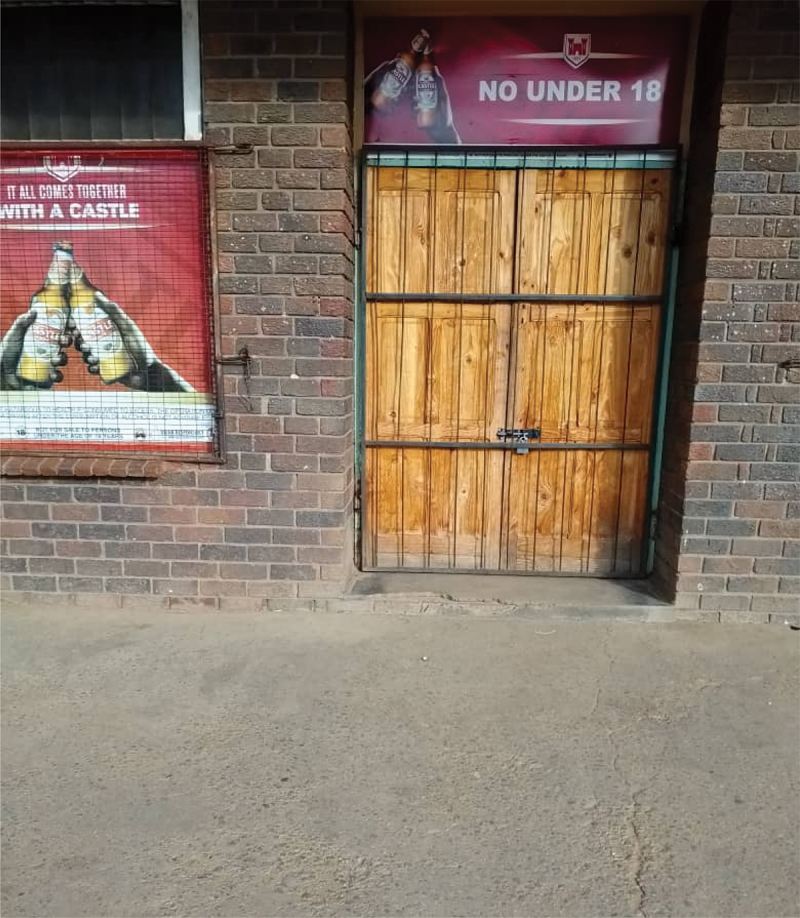
‘Beerhall’.

‘This is a picture (Figure 3) of a beerhall. I took this picture because we get condoms from beerhalls. During lockdown condoms were not easily accessible from beerhalls. Only those who had personal connections with the owner or the barman of the beerhall had access to condoms. They were ordered to close and you could get in trouble if the policemen saw you loitering around.’ Aneni, age 25, live in a small town

Aneni’s account also suggests that some strategies to access condoms involved breaking the law, and further demonstrates the police’s role in enforcing lockdown measures.

Finally, there appears to be certain gender-related circumstances that makes it possible for some women to negotiate access to HIV prevention services from hospitals during lockdown. Jane described how maternal and child health services continued to be available, and that it was through this service provision women would tactically access HIV prevention methods.

Women find it easier because I can just go get my family planning pills or I could be taking my baby for a checkup at the clinic and then get condoms. But when a man goes, what reason will he give? He isn’t sick, he doesn’t need medication, so it is difficult for them … They might be embarrassed” Jane, age 25, live in a subsistence farming area

Similarly, Zendaya, a sex worker, whose occupation is known by nurses in her local clinic, spoke about unfettered access to HIV prevention methods.

‘Condoms are always available in hospitals and they usually give us three boxes of condoms. If I run out I can ask my colleagues … For us as commercial sex workers, it’s not that difficult because we have nurses that assist us to access HIV prevention methods and if any one of us is having challenges we inform them and they come.’ Zendaya, in her twenties, live in a small town

Whilst many of our study participants described experiences and perceptions of difficulties in access to HIV prevention methods, we also noted a resilience, with different tactics adopted to access HIV prevention methods. Communities at risk of HIV did not idly wait for COVID-19 related restrictions to disappear, but took advantage of helpful personal connections (e.g. in health facilities or beer halls) or instigated task-shifting (e.g. community health workers distributing condoms).

## Discussion

Understanding how containment of one infectious disease affects the prevention of another is crucial for future pandemic preparedness. In the present study, we qualitatively explored how COVID-19 and related lockdown measures were experienced and perceived to interrupt access to HIV prevention methods and services in Manicaland Province, Zimbabwe. In our study, almost all participants described challenges to access and spoke about HIV prevention services being temporarily unavailable, either because they were de-prioritised or because of stock-outs. In this way, their experiences of interruption in access to HIV prevention services resonate with international research findings [4,5]. Participants who engaged in transactional sex, and who were known to healthcare providers as female sex workers, reported being prioritised and experiencing no interruption to access, reflecting other research in Zimbabwe [14] and South Africa [13]. Although, as far as we know from our work with local health facilities, there were no issues with stock-outs of HIV prevention methods in Manicaland province. Yet, this perception permeated our data. Other research in Manicaland has noted very strong perceptions of the COVID-19 pandemic resulting in medicine stockouts [20].

This study adds insight into how COVID-19 lockdowns manifested in local and resource-constrained settings, with implications for access to HIV prevention methods. The closure of local beerhalls, a place where condoms are frequently accessed, and restrictions in movement beyond a 5 kilometer radius, meant that access to HIV prevention through essential services, such as pharmacies and health facilities, was interrupted. These observations may go some way to explain the evidence of reduced access to condoms found during the COVID-19 pandemic in neighbouring South Africa [10]. Our research also points to local ingenuity and adaptation in maintaining access to condoms, such as capitalising on personal connections, task-shifting of condom distribution to community health workers or accessing HIV prevention through services that were prioritised during the lockdown (e.g. maternal and child health). Although these ingenuities manifest very differently from the telemedicine adaptations noted internationally [8,9], they provide further insights for understanding the resilience of local health systems and communities affected by HIV in times of crisis.

While many of the barriers discussed in this article were temporary, our findings also point to two potential long-term consequences. While some participants claimed they would abstain from sex as long as HIV prevention methods were inaccessible, others said they had unprotected sex during this period as a direct result of inaccessible HIV prevention methods, heightening their risk of HIV acquisition. While UNAIDS has stated that COVID-19’s impact on HIV services is less severe than feared [21], findings, such as ours, support modelling studies that have calculated the potential devastating impact of interruptions to HIV treatment and prevention services on HIV incidence [22]. Furthermore, the COVID-19 pandemic and related lockdowns have upturned the livelihoods of many people in Manicaland as elsewhere in the world. This article has illustrated how this may introduce poverty-related barriers to accessing HIV prevention methods. However, it may also influence the risk of acquiring an HIV infection. Previous work in Zimbabwe has noted how economic downturns drive high-risk sexual practices [23].

Despite these insights, the analysis has limitations. First, the analysis is based on a small number of people from different settings. Despite their geographical spread, representing different urban and rural contexts, their views and perspectives on COVID-19 were surprisingly homogenous. Most had received a vaccine, and none expressed doubt about COVID-19. Second, this paper covers a period spanning two lockdowns with a period of lower infection rates and somewhat eased control measures in between. Vaccinations were also introduced at the end of the study. The effects of COVID-19 and associated lockdowns on access to HIV prevention may be changing over time (e.g. as dominant variants change, and vaccination coverage increases). The current findings do not provide information on how long-lasting the observed changes will be. However, a strength of the (longitudinal) study design is that these may be seen in subsequent activities with this qualitative research cohort. Third, findings are based on participants’ perceptions; some perceptions of gaps in service availability (for example) may not reflect the actual situation with these services.

## Conclusion

Early and decisive actions to prevent the spread of SARS-CoV-2 were said to affect our participants’ access to HIV prevention in multiple ways. They described both supply side disruptions to the delivery of HIV prevention services and lockdown-induced barriers (e.g. restrictions in movement and fear of COVID-19) to demand. The disruptions were temporary, but long enough to catalyse local responses to the demand for condoms. Despite instances of local adaptation, our study alludes to significant gaps in the pandemic response capacity of HIV prevention services (apart from services for female sex workers) in eastern Zimbabwe. The downstream consequences of the COVID-19 restrictions may ultimately contribute to the reversal of hard-won gains in HIV prevention. This article offers insights into two interrelated critical pathways to attenuate the collision of ongoing and future pandemics. First, HIV services need to be better prepared for future pandemics. This includes developing differentiated service delivery models that can be implemented rapidly in response to restrictions in access to HIV services. This may include elevating the role of community health workers in making essential HIV prevention methods locally available. Second, measures to curb future pandemics must be commensurate with the magnitude of threat. This requires analytical attention to the downstream effects of colliding pandemic prevention measures.

## Paper context

The past couple of years have seen the colliding of two global pandemics: AIDS and COVID-19. Growing evidence suggests that progress against HIV has not only stalled during the COVID-119 pandemic but actively lost ground. This paper provides insight into how people at risk of HIV have experienced this pandemic collision. Focusing on access to HIV prevention methods during lockdown, the findings both inform future pandemic preparedness responses and detail resilience of communities at HIV risk.
